# Nanostructured Metal Oxide-Based Acetone Gas Sensors: A Review

**DOI:** 10.3390/s20113096

**Published:** 2020-05-30

**Authors:** Vahid Amiri, Hossein Roshan, Ali Mirzaei, Giovanni Neri, Ahmad I. Ayesh

**Affiliations:** 1 Department of Materials Science and Engineering, Shiraz University of Technology, Shiraz 71555-13876, Iran; vamiri1365@gmail.com; 2 School of Electrical and Computer Engineering, Shiraz University, Shiraz 51154-71348, Iran; hossein.roshan@shirazu.ac.ir; 3 Department of Engineering, University of Messina, C.da Di Dio, I-98166 Messina, Italy; 4 Department of Math., Stat. and Physics, Qatar University, Doha P.O. Box 2713, Qatar; 5 Center for Sustainable Development, Qatar University, Doha P.O. Box 2713, Qatar

**Keywords:** acetone, gas sensors, metal oxide-based sensor, sensitivity, selectivity, sensing mechanism

## Abstract

Acetone is a well-known volatile organic compound that is widely used in different industrial and domestic areas. However, it can have dangerous effects on human life and health. Thus, the realization of sensitive and selective sensors for recognition of acetone is highly important. Among different gas sensors, resistive gas sensors based on nanostructured metal oxide with high surface area, have been widely reported for successful detection of acetone gas, owing to their high sensitivity, fast dynamics, high stability, and low price. Herein, we discuss different aspects of metal oxide-based acetone gas sensors in pristine, composite, doped, and noble metal functionalized forms. Gas sensing mechanisms are also discussed. This review is an informative document for those who are working in the field of gas sensors.

## 1. Introduction

As a general rule, chemical compounds containing at least one carbon (C) and one hydrogen (H) atoms in their molecular structure are called organic compounds [1]. They are referred as volatile organic compounds (VOCs) when they turn volatile at ambient temperature [2]. As an important member of VOCs, acetone (CH_3_COCH_3_; IUPAC name is propanone) is a widely used substance [3].

**Figure 1:**
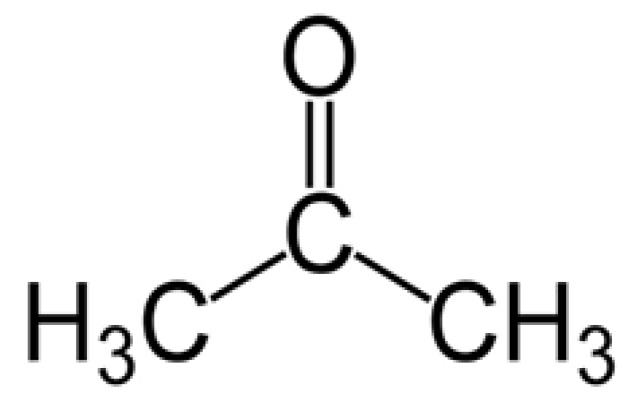
Chemical structure of acetone.

Acetone (see the molecular structure in Figure 1) has a molecular weight of 58.08 g/mole, density of 0.79 g/cm^3^ at 20 °C, and an intense odor, and it is an extensively used solvent in industry and is also found in many common domestic commodities. However, it can be easily inhaled, resulting in serious effects on human health [4]. Acetone concentrations higher than 173 ppm can severely affect the central nervous system and damage important organs of the body [5]. Furthermore, damage to eyes and nose are other effects of long-term exposure to acetone [6]. Accordingly, the occupational threshold limit value for acetone has been set to 250 ppm, considering an 8 h time weighted average [7]. Along with the negative effects to human body, it is a flammable substance with low explosive limit (LEL) of 2.6% and upper explosive limit (UEL) of 12.8% [8].

VOCs are generated either from inside the body (endogenous VOCs) or from external sources such as food ingestion and environmental exposure (exogenous VOCs). The exhaled human breath comprises ~3500 different VOCs [9] and the analysis of VOCs in breath gas may become a promising non-invasive tool and simple health check method that can be conducted both at home and in a medical facility for medical diagnosis and for monitoring the success of therapy [10,11]. For example, breath analysis can be used for the early diagnosis of diseases, such as lung cancer [12], congestive heart failure [13], diabetes [14], and asthma [15]. In addition, high concentration of hydrogen gas in the breath shows a trace of small intestinal bacterial overgrowth (SIBO) in patients suffering from symptoms, such as nausea, bloating, vomiting, diarrhea, malnutrition, weight loss, and malabsorption [16]. Moreover, as ammonia concentration drops after dialysis, a highly sensitive ammonia sensor can be used for a real-time and low-cost breath ammonia sensor for the daily tracking of hemodialysis patients [17]. Acetone is considered an important type-1 diabetes biomarker, and it is reported that the exhaled breath of diabetic people contains a higher concentration value of acetone (>1.8 ppm) compared to healthy people (0.3–0.9 ppm) [18]. Therefore, monitoring breath acetone can be considered as a useful way to follow patients on a prescribed diet regime, as well as to monitor diabetic patients [19]. Furthermore, there is a correlation between acetone and blood glucose level, and thus, its monitoring can be used for insulin management [20].

Concentration of VOCs can be measured using standardized methods such as gas chromatography mass spectrometry (GC-MS) [21], high-performance liquid chromatography [22], and proton transfer reaction mass spectrometry (PTR-MS) [23]. These techniques have high sensitivity and precision for detection of various VOCs. Nevertheless, they are bulky, complex, expensive, time consuming, and need skilled operators for monitoring gases [24]. Therefore, there is a need for small, portable, and fast dynamic devices that can easily detect acetone vapor [25,26].

Gas sensors are electrical devices that can produce an electrical signal in the presence of target gases [27]. A practical gas sensor must be highly sensitive, selective, stable, and fast, with low prices, and requires low operational power [28]. Furthermore, gas sensors should have a high signal-to-noise ratio (S/N), which indicates the relative gas signal intensity over noise intensity. The electrical noise of gas sensors can be determined by measuring the average resistance fluctuation before introduction of target gas. Generally, a low noise level can be induced by a high electrical conductivity [29]. It is accepted that an S/N value of three is needed for estimating the limit of detection (LOD) [30]. LOD is defined as three N/S, where N stands for the root mean square noise and S refers to the slope of the calibration curve of gas sensor [31].

So far, different gas sensors such as field-effect transistors (FETs) [32], optical [33], electrochemical [34], surface acoustic wave [35], cataluminescence [36], microwave-based [37], quartz crystal microbalance [3], microcantilever [38] mixed potential [39,40], photonic waveguide [41], and resistive-based [42,43] have been introduced in the literature.

Among the different gas sensors, resistive-based gas sensors are highly popular due to simple operation principle, small size, high sensitivity, and low cost [44]. Resistive-based gas sensors sense a surrounding gas/gas mixture by changing its electrical resistance and often are fabricated from metal oxides [45]. Brattain and Bardeen first reported the modulation of Ge electrical properties in the presence of a gas [46]. Later, Heiland also reported this effect for ZnO [47]. In 1962, Prof. Seiyama demonstrated the first real metal oxide-based gas sensor using a thin ZnO film for the detection of propane [48]. Then, commercialization was demonstrated for the SnO_2_-based gas sensors [49]. Nowadays, metal oxide-based gas sensors are widely used for detection of more than 150 toxic gases [50,51], and many companies such as Figaro, Winsen, AMS (ScioSense), City Technology, SGX Sensortech, and IDT (Synkera) are offering many types of those sensors.

Previously, Mirzaei et al. [1] presented a comprehensive review paper about nanostructured gas sensors for detection of VOCs. In their review paper, they discussed different VOC gas sensors, however, less attention was paid to acetone. Joshi et al. [52] presented a review paper discussing room temperature chemiresistive gas sensors. However, in their review paper, they only focused on room temperature gas sensors and in addition to acetone, they discussed other types of gases. Alizadeh et al. [53] presented a good and comprehensive review paper about acetone gas sensors. However, they have not focused on metal oxide gas sensors, but all other types of gas sensors are discussed. Masikini et al. [9] recently have published a good review paper about breath acetone sensing by metal oxides. In present review paper, our discussion is focused only on metal oxide-based sensors for acetone sensing. Furthermore, we also dedicated more attention on the acetone sensing mechanism of these typology of gas sensors.

## 2. Metal Oxide-Based Gas Sensors: Introduction, Design, and General Mechanism

Metal oxide-based gas sensors are often fabricated by coating a sensing layer over an insulating substrate and then providing it with interdigitated electrodes [54]. A heater is also included in many sensors in order to raise the temperature of the gas sensor up to 500 °C [55]. Micromachined gas sensors can be used for attaining high temperatures [56]. Furthermore, they are fabricated from nanoscale materials, which have a high surface area and high reactivity to target gases [57,58]. In addition, it is well known that morphology is one of the most important factors affecting the gas sensing performance. Therefore, different morphologies, such as nanoparticles [59], nanorods [39], hollow spheres [60], nanowires (NWs) [61], hierarchical [62], porous spheres [63], flowers [64], and dumbbell-like [65] morphologies have been reported for acetone or other gas sensing studies.

The general mechanism of sensing for metal oxide-based acetone gas sensors is as follows. Initially, in air, due to high electron affinity of oxygen molecules (0.43 eV [66]), they get adsorbed on the surface of the sensor and take electrons from the sensing layer. Accordingly, depending on the operating temperature, different oxygen ion species will be available on the surface of gas sensors as demonstrated by the following equations [67]:

(1) $$O_{2}\left(g\right)\to O_{2}\left(ads\right)$$

(2) $$O_{2}\left(ads\right)+e^{-}\to O_{2}^{-}\left(ads\right)$$

(3) $$O_{2}\left(ads\right)+e^{-}\to O_{2}^{-}\left(ads\right)$$

(4) $$O_{2}^{-}\left(ads\right)+e^{-}\to 2O^{-}$$

(5) $$2O^{-}+e^{-}\to O^{2-}$$

The tested gas species depend on the sensing material and sensing temperature. However, generally, molecular ions are stable below 150 °C, while other forms are stable at higher temperatures [68,69]. Due to abstraction of electrons and depending on the n-type or p-type nature of gas sensor, an electron depletion layer or a hole accumulation layer, respectively, will be generated on the surface of gas sensor. Since the majority of charge carriers in n-type sensors are electrons and those in p-type gas sensors are holes, the resistance of n-type sensors increases relative to vacuum, while the resistance of p-type gas sensors decreases relative to vacuum. When acetone gas is injected into a gas test chamber, it will react with oxygen ions that were already adsorbed and electrons will be released as follows [70,71]:

(6) $${\left(CH_{3}COCH_{3}\right)}_{g}\to {\left(CH_{3}COCH_{3}\right)}_{ads}$$

(7) $${\left(CH_{3}COCH_{3}\right)}_{ads}+8O^{-}\to 3CO_{2}+3H_{2}O+8e^{-}$$

The released electrons are injected in to the sensing layer and the width of electron depletion layer of an n-type sensor or hole accumulation layer of a p-type sensor is decreased. Accordingly, the resistance of n-type sensors decreases and that of p-type gas sensors increases upon exposure to acetone. The response of gas sensor to acetone gas, which is a reducing gas, is usually defined as R_a_/R_g_ for n-type gas sensors and R_g_/R_a_ for p-type gas sensors, where R_a_ is the resistance of gas sensor in the presence of air and R_g_ is the resistance of gas sensor in the presence of acetone gas.

**Figure 2:**
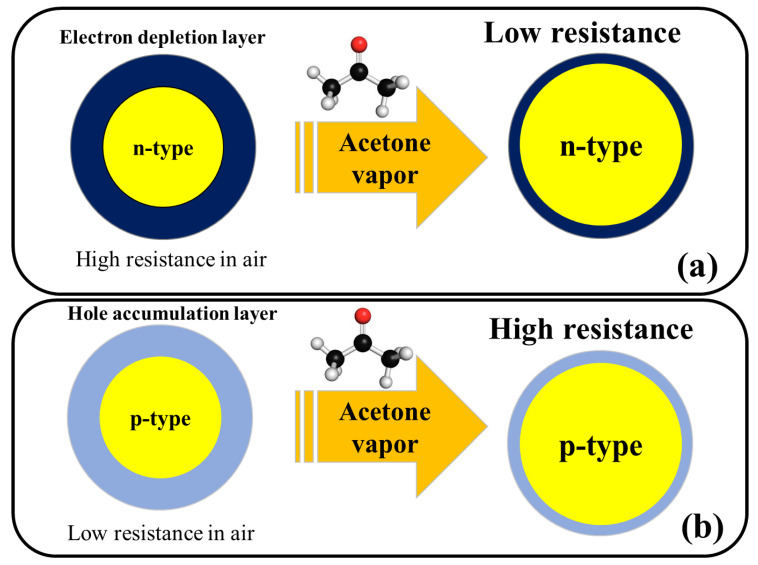
Schematic illustration of acetone sensing mechanism in (**a**) n-type metal oxides and (**b**) p-type metal oxides.

Figure 2 shows the general acetone sensing mechanism for pristine n-type and p-type metal oxides. For noble metal-decorated or heterojunction metal oxide-based gas sensors, in addition to above mechanism, formation of Schottky contacts, formation of n–n, n–p, or p–p heterojunctions, and catalytic effects of noble metals are other mechanisms which can further enhance the response of gas sensor and will be explained in the subsequent sections of this article.

In general, nanostructured materials such as 0-dimensional, 1-dimensional, and 2-dimensional metal oxides have higher sensitivity relative to their bulk counterparts. In fact, such materials not only have a high surface area which greatly enhances the response of gas sensors due to offering of more adsorption sites for the target gas molecules but they also have many contact points which act as potential sources for the resistance modulation. Another advantage of nanostructured metal oxides stems is from the fact that when the size of materials reaches to the Debye length (*λ_D_*), adsorption of target gas molecules on the surface of sensor leads to great modulation of electrical resistance, leading to the appearance of a high sensing signal [72].

## 3. Pristine Acetone Gas Sensors

In 1991, bismuth ferrite (BiFeO_3_) nanoparticles (NPs) were studied for sensing applications and it was reported that sensing performance was related to the chemical composition [73]. Moreover, recently, BiFeO_3_ NPs were used for acetone sensing and they showed a response (R_g_/R_a_) of 10–12 ppm acetone gas at 350 °C with a fast response time of 25 s and a recovery time of 17 s [74]. In another study, p-type praseodymium ferrite (PrFeO_3_) was used for sensing studies. Electrospun hollow PrFeO_3_ nanofibers with a high surface area of 33.74 m^2^/g showed a high response (R_g_/R_a_) of 141–200 ppm acetone at 180 °C [75].

**Figure 3:**
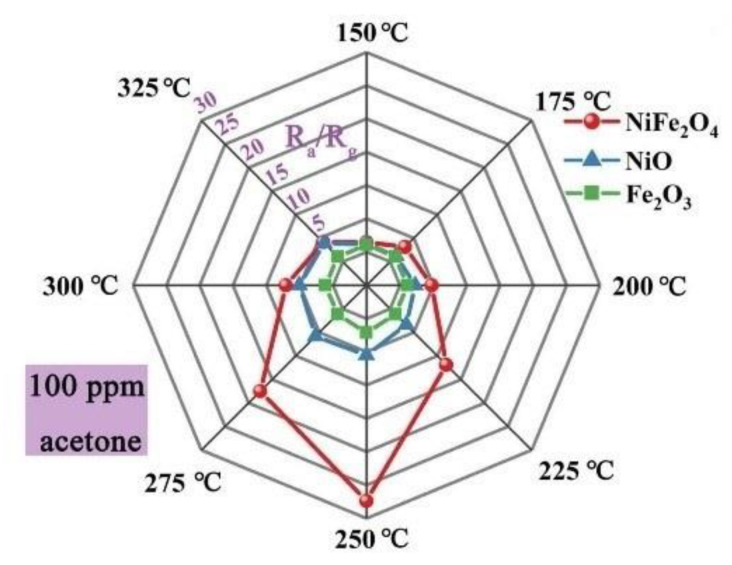
Radar graph showing responses of NiFe_2_O_4_, NiO, and Fe_2_O_3_ gas sensors at various temperatures to 100 ppm acetone. [18]. Reprint permission was obtained from Elsevier.

Semiconducting spinel ferrites (AFe_2_O_4_), in which the transition metal cation “A” was included into the lattice of the parent structure of (Fe^2+^Fe_2_^3+^O_4_) [76,77,78,79], had environmental friendliness, low cost, and excellent stability [80,81,82]. Therefore, they were studied for acetone sensing applications [83,84]. For example, super fine porous NiFe_2_O_4_ spheres were fabricated using a solvothermal method. The surface area of the product was 20.04 m^2^/g with an average pore size of ~8.9 nm. The fabricated sensor revealed a high response (R_g_/R_a_) of 27.4–100 ppm acetone with a fast response time of 2 s at 250 °C, as shown in Figure 3. The sensor showed a moderate selectivity to acetone. In addition, the response to acetone was significantly decreased with an increase of relative humidity, which limits its real-world applications [18].

In another study, the effects of surface area and morphology on acetone sensing was studied by synthesis of ZnCo_2_O_4_ nanotubes, ZnCo_2_O_4_ nanosheets, and multishelled ZnCo_2_O_4_ yolk–shell spheres. It was reported that the multishelled ZnCo_2_O_4_ yolk–shell spheres exhibited the highest response to acetone among all gas sensors. It showed a response (R_g_/R_a_) of 38.2–200 ppm acetone at 200 °C. High response of the optimal sensor was related to the special morphology of the gas sensor with improved accessibility of gas to the surface of the sensor, small crystal size, as well as high surface area (74.8823 m^2^/g) of the gas sensor. In addition, fast dynamics was attributed to the porous nature of the gas sensor. In comparison, the surface area of ZnCo_2_O_4_ nanotubes and ZnCo_2_O_4_ nanosheets were reported to be 10.027 m^2^·g^−1^ and 60.514 m^2^·g^−1^, respectively [85]. Therefore, a lower gas response was reported for these morphologies. However, due to relatively high response to ethanol, this gas sensor is not useful for acetone sensing in exhaled breath analysis.

Fe_2_O_3_ with a bandgap of 2.1 eV is the most stable iron (III) oxide and has been known as an excellent n-type material for gas sensing to detect various gases in pristine [86] or composite form [87]. It has many fascinating properties such as low cost, environmentally friendly nature, and excellent electrical properties [86]. A novel Fe_2_O_3_ foam was synthesized by a facile and simple sol–gel autocombustion route. The novel morphology of the Fe_2_O_3_ foam enhanced the sensing properties of the gas sensor at 300 °C, where it showed a response (R_a_/R_g_) of 11.60–100 ppm acetone with fast response and recovery times of 4 and 10 s, respectively. The unique micro/nanoholes provided more active sites and provided a rapid pathway for the transportation/diffusion of the acetone gas, resulting in enhanced response to acetone vapor [88]. In another study dealing with pristine Fe_2_O_3_ NPs, a response (R_a_/R_g_) of 9–100 ppm acetone at 340 °C was reported. However, its selectivity was not acceptable, thus limiting its utilization for real-world applications [89].

**Figure 4:**
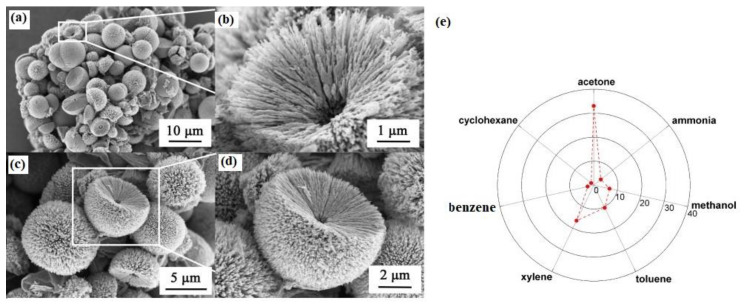
(**a**–**d**) Scanning electron microscopy images of hierarchical ZnO spheres. (**e**) Selectivity graph of ZnO spheres gas sensor at 230 °C [90]. Reprint permission was obtained from Elsevier.

ZnO is also another popular metal oxide for gas sensing studies. Hierarchical nanostructured ZnO spheres were fabricated through a solvothermal method (Figure 4a–d). Dandelion-like sphere ZnO revealed a response (R_a_/R_g_) of 33–100 ppm acetone gas at 230 °C with short response time of 3 s. Once ZnO is exposed to the gas of acetone, further acetone molecules could react with the absorbed oxygen species. Additionally, the gas sensor showed high selectivity to acetone gas, as shown in the polar graph of Figure 4e. The high selectivity was related to the larger dipole moment of acetone (2.88 D) compared to other gases. Accordingly, acetone has a higher chance of being adsorbed on the polar (002) facets of ZnO (compared with other gases), resulting in a good selectivity sensor to acetone gas [90].

In another study dealing with ZnO, Zeng et al. prepared ZnO nanorods (NRs) for gas sensing studies [91]. It is found that the ZnO nanorod thin thin-film sensor revealed the highest gas sensing of 33–100 ppm acetone gas at 300 °C with the response time shorter than 5 s. Modulation of depletion layer along length of ZnO NRs was the main cause of resistance modulation in ZnO NRs.

**Figure 5:**
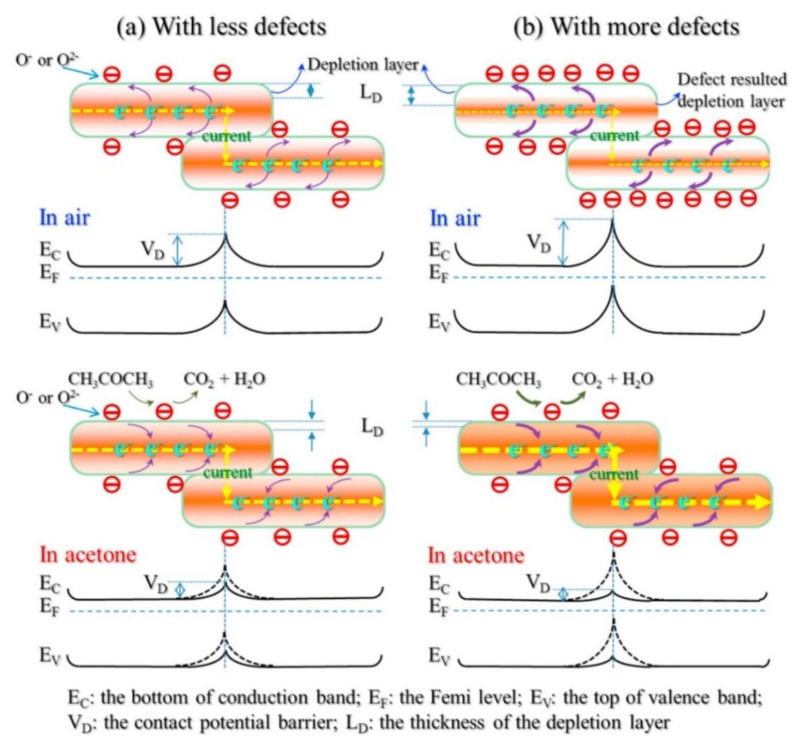
Gas sensing mechanism of ZnO nanosheet with different amount of defects to acetone gas: (**a**) with less defects (**b**) with high defects [92]. Reprint permission was obtained from Elsevier.

ZnO nanosheets were directly synthesized using a precipitation method. The specific surface area of synthesized nanosheets calcined at 200, 400, and 600 °C were 23.88, 21.22, and 8.42 m^2^/g, respectively [92]. In addition, photoluminescence and XPS studies revealed higher amount of oxygen vacancies in the ZnO nanosheet calcined at 200 °C. Based on the acetone sensing results, the highest gas response was observed in ZnO nanosheet calcined at 200 °C, where they showed a response (R_a_/R_g_) of 110–200 ppm acetone at optimal sensing temperature (300 °C). Enhanced gas sensing in this sample was due to higher surface area and presence of the highest amounts of oxygen vacancies. Figure 5 shows the sensing mechanism of ZnO nanosheet. When the amount of oxygen vacancies, which act as a favorable adsorption site for acetone gas, is high, they lead to formation of potential barriers with the higher height (Figure 5b), relative to the sensors with lower oxygen vacancies (Figure 5a), in the contact area between ZnO nanosheets. As a result, upon exposure to acetone gas, for the sensor with higher oxygen defects more change in the potential height between ZnO nanosheet occur, compared to one with lower amount of oxygen vacancies, leading to higher gas response in the sensor with higher oxygen vacancies.

Two-dimensional ZnO nanosheets with high surface area of 38.3 m^2^/g were synthesized using a hydrothermal route for acetone sensing studies [93]. At optimal sensing temperature of 360 °C, the sensor showed a response (R_a_/R_g_) of 33–50 ppm acetone gas.

As a good candidate for gas sensing material, Co_3_O_4_ materials have attracted widespread attention due to their low cost and relatively good sensitivity [94]. Cobalt oxide (Co_3_O_4_) with an intrinsic p-type property (E_g_ = 1.6–2.2 eV) has been studied for low temperature acetone sensing [95]. The high performance for acetone detection can be attributed to the unique structure of Co_3_O_4_ crossed nanosheet arrays were synthesized by a hydrothermal route [95]. The sensor showed a response (R_g_/R_a_) of ~36.5–1000 ppm acetone gas at 111 °C. The high performance for acetone detection can be attributed to the unique structure of Co_3_O_4_ arrays, where they formed ordered array on the substrate with open structure. This led to a high surface area of Co_3_O_4_ arrays (83 m^2^/g). Furthermore, it had a mesoporous structure, enhancing the penetration of oxygen and acetone gas molecules in the depth parts of gas sensor.

In an interesting study, Co_3_O_4_ nanostructures with two different morphologies, namely, nanocubes and nanospheres, were prepared using a hydrothermal method [96]. The response (R_g_/R_a_) of Co_3_O_4_ nanocubes gas sensor to 500 ppm acetone was 4.88 at 240 °C. In fact, it was three times higher than that of Co_3_O_4_ nanospheres at 240 °C.

For Co_3_O_4_ nanocubes, only (100) crystal facets, composed of active surface Co^2+^ cations were exposed to acetone gas. Tetrahedron-coordinated Co^2+^ ions could easily be oxidized to Co^3+^ in air, and upon further exposure to acetone vapor, they could be reduced to Co^2+^ ions. Moreover, for p-type sensing materials, grain boundaries were a channel with low resistances for hole movement. Accordingly, Co_3_O_4_ nanocubes with more contact areas (grain boundaries) among the nanocubes provided more transfer paths for holes, leading to a higher response of Co_3_O_4_ nanocubes relative to nanospheres.

Table 1 summarizes the acetone sensing performance of metal oxide-based gas sensors reported in the literature. Sensing temperature is generally high, which leads to high power consumption by the gas sensor.

Also, it should be noted that the reported response values in Table 1 greatly depend on the concentration of acetone, sensing temperature, sensor morphology, surface conditions, and so on. Therefore, when comparing response values, concentration of acetone vapor and other related parameters should be mentioned.

## 4. Doped Acetone Gas Sensors

### 4.1. Binary Metal Oxide Gas Sensors

Doping is a widely used strategy to enhance acetone sensing properties [99,100]. In an interesting study, the bare and W-doped NiO hierarchical hollow spheres were synthesized by a hydrothermal method. The sensor with 4.0 at% of W-doping showed a response (R_g_/R_a_) of 198.1–100 ppm acetone at 250 °C, while the response of bare sensor was 139 times lower. Higher response of doped sensor was related to the lower crystalline size as a result of W-doping, higher surface area (217.2 m^2^ g^−1^), and higher concentrations of hole carriers due to W-doping, which created additional holes in the NiO [101].

2D Sn-doped ZnO ultrathin nanosheet networks were synthesized by a hydrothermal method [102]. At 320 °C, which was reported as the optimal sensing temperature, the sensor showed a response (R_a_/R_g_) of 5.6–50 ppm acetone gas. Role of Sn-doping was attributed to the creation of more defect in crystal by substitution in ZnO crystal structure.

La_2_O_3_ is rarely used for acetone sensing studies. Xu et al. [103] synthesized bead-like 1 wt% La_2_O_3_-doped ZnO NFs through electrospinning for acetone sensing studies. The gas sensor showed a response (R_a_/R_g_) of 48–100 ppm acetone gas at 340 °C. The excellent gas sensing properties caused by La doping were related to the larger surface area of gas sensor, the increased adsorption capacity of O due to presence of La_2_O_3_, and the useful hetero contacts between n-type ZnO and p-type La_2_O_3_.

Co-doped ZnO NFs were produced through an electrospinning process followed by an annealing process [104]. At optimal sensing temperature of 360 °C, the response (R_a_/R_g_) of 0.5 wt% Co-doped ZnO NFs to 100 ppm acetone was ~16, while that of pristine sensor was only 4.4. Additionally, response and recovery times of optimal sensor were 4 and 6 s, respectively. High response to acetone was due to the high surface area of synthesized NFs and presence of plenty of NF–NF junctions in the netlike structure. Furthermore, Co-doped sensor showed a decreased NF diameter relative to pristine ZnO NFs. Finally, catalytic effect of Co_3_O_4_ led to easy oxidation of acetone, contributing to the sensor signal.

Hollow NFs can provide significantly higher surface area relative to NFs and therefore, it can be expected to have high response to acetone gas. Y-doped SnO_2_ hollow NFs were synthesized through electrospinning technique. The 0.4 wt% Y-doped SnO_2_ hollow NFs showed a response (R_a_/R_g_) of 174–500 ppm acetone gas at 300 °C [105]. High sensing performance to acetone gas was related to the high surface area of gas sensor (29.46 m^2^/g), presence of many small nanograin–nanograin junctions on the surface of SnO_2_ hollow NFs, and catalytic effect of surface “Y” clusters, which enhanced gas response to acetone gas.

Like other rare earth elements, Eu rarely has been studied for gas sensing studies. In a study by Jiang et al. [106], Eu-doped SnO_2_ NFs were produced by electrospinning for acetone sensing studies. The sensor was able to detect even 0.3 ppm acetone, making it a potential candidate for the breath diagnosis of diabetes. When Eu^3+^ ions diffused into the SnO_2_ lattice and substituted Sn^4+^ ions, the mismatch in ionic radius between Eu^3+^ (0.947 Å) and Sn^4+^ (0.69 Å) resulted in the lattice distortion and defects, leading to enhanced gas response. Furthermore, Eu_2_O_3_ was able to accelerate the reactions between acetone molecules and absorbed oxygen species to release electrons to the conduction band, leading to an improved gas response.

In another study, the effect of Au doping in the core–shell structure of SnO_2_/In_2_O_3_ was investigated. SnO_2_/Au-doped In_2_O_3_ core–shell nanofibers (NFs) with a surface area of 69.4 m^2^ g^−^1 were obtained by a coaxial electrospinning technique [107]. The sensor displayed a response (R_a_/R_g_) of 21–100 ppm acetone at 280 °C. Even though the sensor showed a fast response time of 3 s, its recovery time was long (170 s). The high response was attributed to the almost full of electron In_2_O_3_ shell layer due to transfer of electrons to SnO_2_ as well as adsorption by oxygen molecules from air. Accordingly, upon exposure to acetone vapor and release of electrons back to the surface of In_2_O_3_ shell, a significant amount of resistance modulation led to a high response to acetone gas. Furthermore, catalytic effects of Au should not be disregarded. In fact, at a high sensing temperature, oxygen molecules become dissociated on the surface of Au and then, in a spill over process, move to the surface of In_2_O_3_, leading to adsorption of plenty of oxygen ions on the surface of sensor. As a result, within the acetone atmosphere, the reaction between adsorbed oxygen molecules and acetone significantly increases the response of the gas sensor.

Rh is a noble metal with good catalytic activity to acetone gas [108]. Accordingly, the addition of Rh to electrospun SnO_2_ NFs could be a good strategy for acetone sensing enhancement, which has been recently reported by [108]. The grain size of the nanocrystals of SnO_2_ dramatically decreased as a result of Rh doping. At a sensing temperature of 200 °C, the 0.5 mol% Rh-doped sensor showed a response (R_a_/R_g_) of 60.6–50 ppm acetone gas. The doped sensor showed a higher baseline resistance relative to the pristine sensor due to a smaller crystalline size and the replacement of Sn^4+^ by Rh^3+^. Additionally, due to Rh doping, both oxygen vacancies and chemisorbed oxygen ions were greater than that of the pristine gas sensor. The first sensor provided more adsorption sites for acetone gas molecules, while the latter sensor resulted in more reactions between acetone and oxygen molecules. Accordingly, a higher response was resulted for doped gas sensors [109].

In another study, 0.5 at% Ru-doped NiO flower-like spheres exhibited improved sensitivity to acetone at 200 °C. In particular, the sensor showed a negligible decrease of response in humid air. In fact, due to catalytic activity of Ru, both chemisorbed oxygen ions and reaction rates between acetone and adsorbed oxygen increased, leading to decrease of humidity effect on the overall gas response [110].

Porous Pt-doped In_2_O_3_ NFs were synthesized for acetone sensing studies. The Pt-In_2_O_3_ porous NFs showed a very high surface area of 212.3 m^2^·g^−1^. Owing to the catalytic activity of Pt, formation of Pt-In_2_O_3_ Schottky barriers, and the porous structure, it was possible to detect concentrations as low as 10 ppb acetone gas at 180 °C, with fast response and recovery times of 6 and 9 s, respectively. The good selectivity to acetone was attributed to the bond dissociation energy of CH_3_CHO (352 kJ·mol^−1^), which was smaller than that of HCHO (368 kJ·mol^−1^), NH_3_ (452 kJ·mol^−1^), H_2_S (376 kJ·mol^−1^), H_2_ (436 kJ·mol^−1^), C_7_H_8_ (371 kJ·mol^−1^), CH_3_OH (462 kJ·mol^−1^), and CH_3_CH_2_OH (462 kJ·mol^−1^) [111].

WO_3_ is a metal oxide that is semiconductive (n-type with E_g_ = 2.6–3.0 eV) and is popular for sensing studies [112]. In an interesting study, Cr doped, ε-WO_3_ NPs were synthesized for the first time by flame spray pyrolysis. The sensor showed a higher response to acetone than other gases. The ε-WO_3_ had ferroelectric properties and a spontaneous electric dipole moment that was result of the displacement from the center of each tungsten atoms’ [WO_6_] octahedra. The dipole moment of acetone, which was higher than those of ethanol 1.69D, methanol 1.70 D, NO 0.159 D, NO_2_ 0.316 D, NH_3_ 1.471 D, and CO 0.112 D gases [113], led to higher interaction among the ε-WO_3_ surface dipole and molecules of acetone. In another study, Cr_2_O_3_-doped WO_3_-composite thin films were prepared by a sol–gel route. The sensor with 5 mol% Cr_2_O_3_ showed the highest response (R_a_/R_g_) of 9–20 ppm acetone gas at 320 °C. For higher Cr content, the second phase (CrWO_4_) limited the adsorption of acetone gas on the surface of gas sensor. For lower Cr-content, the resistance of sensor was low, leading to low response of gas sensor [114]. For the sensor with 5 mol% Cr_2_O_3_, due to distortion of WO_3_ lattice, it showed the ultimate acentric structure and the least symmetry. Therefore, it revealed the largest dipole moment, and since the dipole moment of acetone gas was higher than other tested gases, a higher interaction among the WO_3_ surface and acetone molecules occurred, resulting in high response of gas sensor.

**Figure 6:**
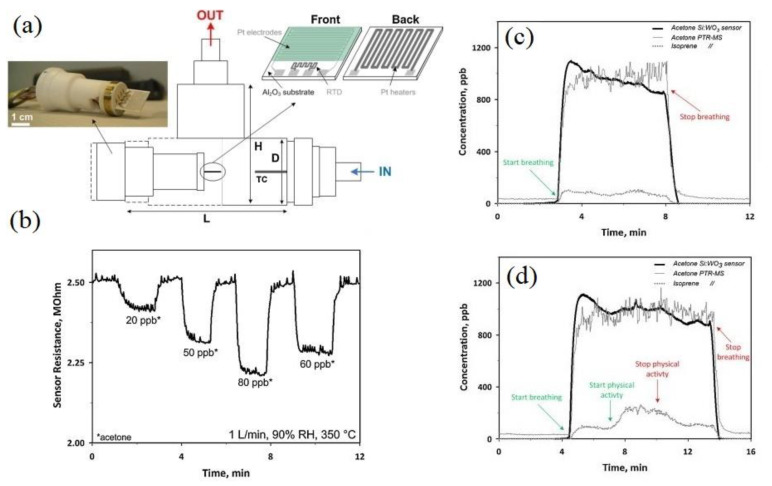
(**a**) Schematic of gas chamber used for breath analysis. (*L* = 75, *H* = 50, and *D* = 18 mm, thermocouple (TC). (**b**) Dynamic resistance curve of 10 mol% Si-doped WO_3_ gas sensor to low concentrations of acetone under 90% RH at 350 °C. (**c**) Dynamic resistance curves of the Si:WO_3_ sensor (thick solid line), acetone (thin solid line), and isoprene (dotted line) gas tested using proton transfer reaction mass spectrometry (PTR-MS) through breathing of a test person (**c**) at rest and (**d**) during physical activity [115]. Reprint permission was obtained from Elsevier.

As previously mentioned, acetone is also an important biomarker of diabetes. In this context, a gas sensor was realized from a 10 mol% Si-doped WO_3_ NPs film for breath analysis (Figure 6a) [115]. For breath acetone analysis, one important interfering VOC is ethanol. The average ethanol concentration in the exhaled breath is ~196 ppb, making it a disturbing VOC during breath acetone measurement. First, the response of the gas sensor to 500 ppb ethanol and 500 ppb acetone was investigated at different sensing temperatures. It was found that at all tested temperatures, the response to ethanol was much lower than that of acetone, demonstrating good acetone selectivity. Moreover, the optimal sensing temperature to ethanol was at ~325 °C, while that of acetone was at 350 °C.

Figure 6b shows a dynamic resistance curve of the gas sensor to low concentrations of acetone gas under 90% relative humidity (RH%) at 350 °C, demonstrating successful detection of parts per billion levels of acetone in simulated breath. Figure 6c,d exhibit the dynamic curves to acetone gas at 90% RH and at 70 mL min^−1^ flow in tidal part of the respiratory cycle of a healthy person at rest detected by the present Si:WO_3_ sensor (thick solid line) and proton transfer reaction mass spectrometry (PTR-MS) for acetone (thin solid line) and isoprene (dotted line), respectively. In both cases, the gas sensor showed an almost similar trend with the PTR-MS. For the person at rest, the response time to human breath and for the 10 mol% Si-doped WO_3_ sensor was of 27 and 28 s, respectively. This demonstrated success of the gas sensor for analysis of acetone in breath analysis.

### 4.2. Ternary Metal Oxide Gas Sensors

P-type LaMnO_3+δ_ (LMO) perovskites oxides have fascinating properties owing to excess oxygen as well as coexistence of Mn^3+^/Mn^4+^ ions with different oxidation states [116]. In order to enhance gas sensing properties of the LMO gas sensor, yttrium-doped LMO NPs (namely, La_0.85_Y_0.25_MnO_3+δ_ NPs) were synthesized via a sol–gel method. It showed a response (R_g_/R_a_) of ~26–500 ppm acetone gas at 300 °C. However, its response to ethanol was almost 5 times lower than that of the pristine gas sensor. Due to doping of Y^3+^ and in order to maintain the charge neutrality, the Mn^4+^/Mn^3+^ ratio was increased, and accordingly, more oxygen species were adsorbed on the surface of gas sensor. Due to the p-type nature of gas sensor, initial resistance of the sensing layer was significantly decreased, and upon exposure of sensor to acetone vapor, the resistance significantly increased, leading to high response of sensing layer to acetone [117].

P-type ytterbium ferrites, with a general formula of YbFeO_3_, have high stability, and their properties can be tuned through metal doping. In an interesting study related to YbFeO_3_, 20 at% Ca-doped YbFeO_3_ (Yb_0.8_Ca_0.2_FeO_3_) was synthesized via a sol–gel process for sensing study. Interestingly, the response to acetone gas was increased in humid air at room temperature. The presence of –OH groups on the surface of the sensor was reported to be the reason for such an increase. However, it seems that more studies are needed to find such a strange behavior of this gas sensor [118].

## 5. Decorated/Loaded Acetone Gas Sensors

**Figure 7:**
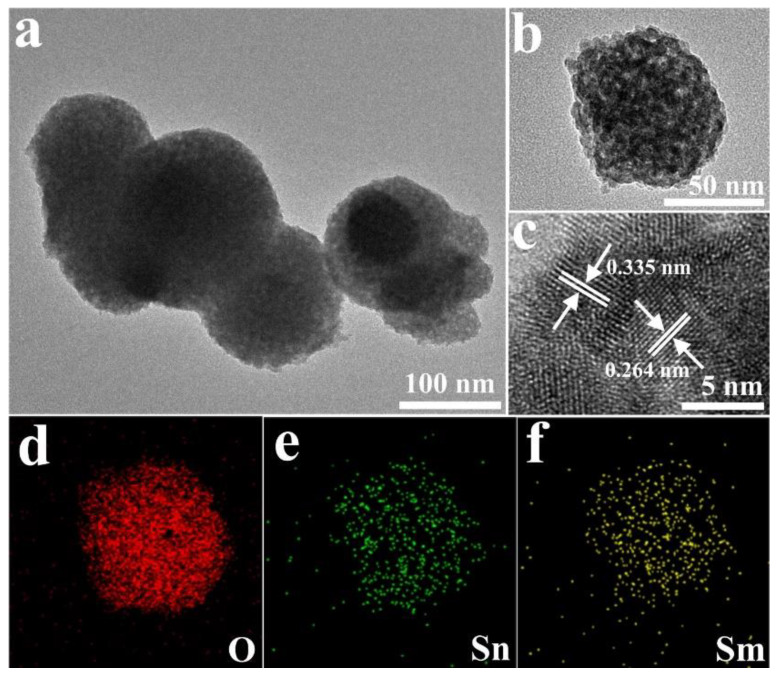
(**a**,**b**) TEM images and (**c**) HRTEM image of 2.5 mol% Sm_2_O_3_-doped SnO_2_. (**d**–**f**) The Result of corresponding elemental mapping analysis [121]. Reprint permission was obtained from Elsevier.

SnO_2,_ is a metal oxide n-type semiconductor (Eg = 3.6 eV) with high stability, low synthesis costs, and high sensing properties due to its high mobility of electrons (160 cm^2^/V·s) [119,120]. SnO_2_ hierarchical structures loaded with Sm_2_O_3_ (0.5, 1, 2.5, and 4 mol%) were synthesized by a hydrothermal method and subsequent isometric impregnation technique (Figure 7). It was revealed that the 2.5 mol% Sm_2_O_3_/SnO_2_ gas sensor showed a response (R_a_/R_g_) of 41.14–100 ppm acetone gas at 200 °C, with a limit of detection of 100 ppb. Upon replacement of Sm^3+^ in SnO_2_ lattice, oxygen vacancies were formed and much higher target gases were adsorbed on the surface of the gas sensor. Furthermore, the catalytic effect of Sm_2_O_3_ was reported to be important for acetone sensing [121].

**Figure 8:**
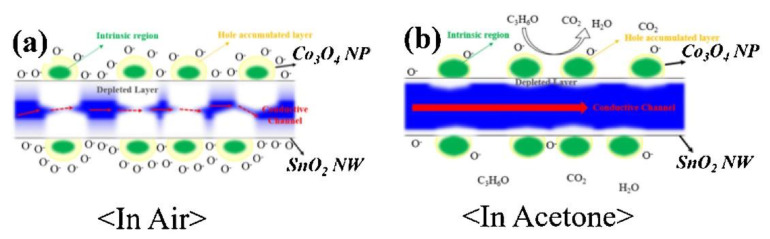
Schematic of acetone sensing mechanism of Co_3_O_4_-loaded SnO_2_ NWs (**a**) in air and (**b**) in acetone [122]. Reprint permission was obtained from Elsevier.

In another study using SnO_2_, p-Co_3_O_4_ loaded-n-SnO_2_ nanowires (NWs) were synthesized by a combination of vapor–liquid–solid (VLS) and sol–gel processes. The gas sensor displayed a response (R_a_/R_g_) of ~62–50 ppm acetone at 300 °C. Enhanced response to acetone vapor was related to the formation of p–n heterojunctions. By attaching p-type Co_3_O_4_ NPs, p–n junctions were formed and electrons in SnO_2_ were transferred to Co_3_O_4_. Accordingly, the width of conduction channel inside of SnO_2_ was significantly decreased, as shown in Figure 8a. Due to the release of electrons back to the surface of gas sensor, the width of conduction channel significantly increased in the acetone atmosphere (Figure 8b), resulting in the improvement of conductivity of the gas sensor [122].

TiO_2_ NPs were decorated on In_2_O_3_ NWs for acetone sensing studies [123]. At 250 °C, the fabricated sensor showed a response (R_a_/R_g_) of ~34–10 ppm acetone which was higher than that of ethanol and other interfering gases. Enhanced sensing response to acetone was due to the changes in the surface depletion layer width and potential energy barrier width of In_2_O_3_ NWs.

Rh_2_O_3_-decorated WO_3_ NFs were synthesized via an electrospinning process for acetone sensing studies [124]. The sensor showed a response (R_a_/R_g_) of 41.2–5 ppm acetone gas at 300 °C. When Rh_2_O_3_-decorated WO_3_ NFs were exposed to target gas, due to reduction of Rh_2_O_3_ NPs, the width of depletion region inside of WO_3_ was decreased, leading to enhanced gas response towards acetone. Furthermore, presence of oxygen vacancies contributed to generation of a high sensing signal.

Not only metal oxides but also noble metals can be decorated on the surface of sensing layers. Different noble metals have been decorated on the sensing layer for acetone sensing studies [125,126]. Hollow porous Fe_2_O_3_ nanocubes were synthesized, and subsequently, Pt NPs were loaded on the surface of Fe_2_O_3_ nanocubes by means of a reduction process. The sensor had a response (R_a_/R_g_) of 25.7–100 ppm of acetone at 139 °C. Both chemical sensitizations and electronic sensitization of Pt had a high contribution to the sensing signal. Oxygen molecules were dissociated on the surface of Pt and subsequently spilt over the surface of Fe_2_O_3_, leading to high adsorption of oxygen ions. The surface of Pt NPs preferentially adsorbs oxygen molecules in air and then spill the oxygen species over to the Fe_2_O_3_, owing to the catalytic promotion of Pt. Furthermore, the electrons were transferred from Fe_2_O_3_ to Pt, and Schottky barriers were formed in air. In an acetone atmosphere, the height of the Schottky barrier significantly decreased, leading to an enhanced response to acetone. Furthermore, it was reported that in air, some PtO_x_ can be formed and the resultant p–n heterojunctions can enhance resistance modulation in the presence of air [127].

In another study, the effect of codecoration on the sensing response to acetone was studied. A PdAu-decorated SnO_2_ three-dimensional nanosheet gas sensor revealed a response (R_a_/R_g_) of 6.5–2 ppm at 250 °C with fast response time of 5 s and recovery time of 4 s. Additionally, the sensor displayed a negligible difference in the response to acetone vapor in the presence of high level (94%) relative humidity. Apart from generation of Schottky barriers between Au-Pd and SnO_2_, both Au and Pd had a high oxygen dissociation capability and created activated oxygen species that were spilled over the SnO_2_. In addition, synergistic catalytic effects of the Pd/Au NPs promoted the reactions between acetone and adsorbed oxygen species. Furthermore, due to unique structure of the gas sensor and porous morphology, diffusion of target gases was facilitated inside the gas sensor, leading to enhanced dynamics of gas sensor [128].

## 6. Composite Acetone Gas Sensors

In the literature, there are many studies related to acetone sensing properties of composite materials. This is due to the fact that in composite materials, plenty of heterojunctions can form, leading to significant modulation of electrical resistance [64]. However, in this review paper, we just report the results of the most interesting acetone gas sensors in terms of design or gas sensing performance.

Electrospun Pt@In_2_O_3_ core–shell composite NWs were prepared for acetone sensing studies [129]. The sensor showed a highly improved response, short response and recovery time of 14 and 16 s, respectively, for 1 ppm of acetone, and high selectivity and stability compared with a sensor based on pristine In_2_O_3_ NWs due to the increase in surface resistance and the presence of heterojunctions. In addition, detection limit was low as 10 ppb, which was much lower than the concentration level of 1.8 ppm in the exhaled breath of diabetic patients. The influence of the large amount of moisture was greatly weakened by using the molecular sieve as a moisture filter layer, leading to much improved sensitivity to acetone in clinical sample detection.

WO_x≤3_ oxide has different nonstoichiometric phases, namely, WO_2_, WO_2.72_, WO_2.8_, and WO_2.9_. In general, these phases have a lot of oxygen vacancies, which promote the adsorption of target gases on the surface of gas WO_x≤3_-based gas sensors [130]. In addition, MXenes are a new class of 2D transition metal of carbides/nitrides, that are rich with functional groups such as –O, –OH, and –F, which are beneficial for gas sensing studies [131]. In this regards, a series of WO_2._72(W_18_O_49_)/Ti_3_C_2_T_x_ composites were solvothermally prepared for sensing studies towards acetone [132]. The W_18_O_49_ nanorods were distributed on the surface of Ti_3_C_2_T_x_ nanosheets, leading to an increase of adsorption sites relative to pristine sensors. The W_18_O_49_/Ti_3_C_2_T_x_–2% sensor showed a response (R_a_/R_g_) of 4.2–5 ppm acetone gas at 300 °C, which was higher than other gas sensors with different amounts of Ti_3_C_2_T_x_ as well as pristine gas sensors. First, due to presence of functional groups on the surface of Ti_3_C_2_T_x_, the acetone molecules were effectively adsorbed on the surface of the gas sensor. Second, due to metallic nature of for the Ti_3_C_2_T_x_, heterojunctions were creased on the interfaces between Ti_3_C_2_T_x_ and W_18_O_49_, and in the acetone atmosphere, the modulation of heterojunction potential barrier led to enhanced sensing response. Third, for the compositions with a higher amount of Ti_3_C_2_T_x_, some –F groups were attached to the surface, leading to decrease of gas response. Finally, too much Ti_3_C_2_T_x_ caused the stacking of the nanosheets, and this decreased the adsorption sites of gas sensor.

**Figure 9:**
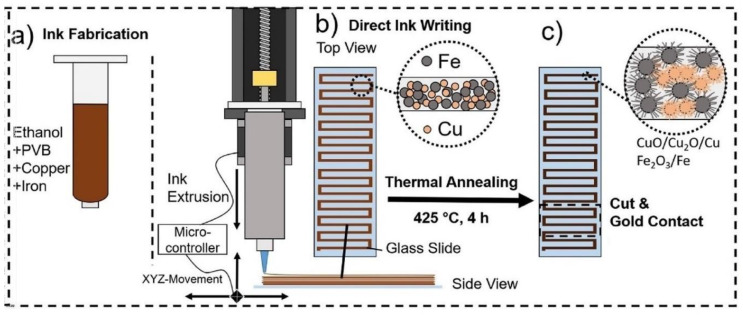
(**a**–**c**) Different steps for preparation of CuO/Fe_2_O_3_ gas sensor using direct ink writing (DIW) method [134]. Reprint permission was obtained from Elsevier.

CuO with p-type sensing properties has been used for sensing studies due to its low synthesis cost and catalytic effects [133]. A very low power consumption gas sensor was prepared using Fe_2_O_3_/CuO-based nanostructures which were fabricated by direct ink writing (DIW) on top of the surface of an insulating (glass) substrate (Figure 9). Due to the presence of copper oxides and iron oxides, many heterojunctions were produced on the surface of glass substrate. The power consumption of gas sensor at 300 °C was only 0.26 μW, and the gas sensor showed a response of 50% to 100 ppm acetone vapor. Formation of Fe_2_O_3_/CuO heterojunctions in air and subsequent modulation of potential barriers in acetone vapor were the main reasons for acetone sensing mechanism [134].

**Figure 10:**
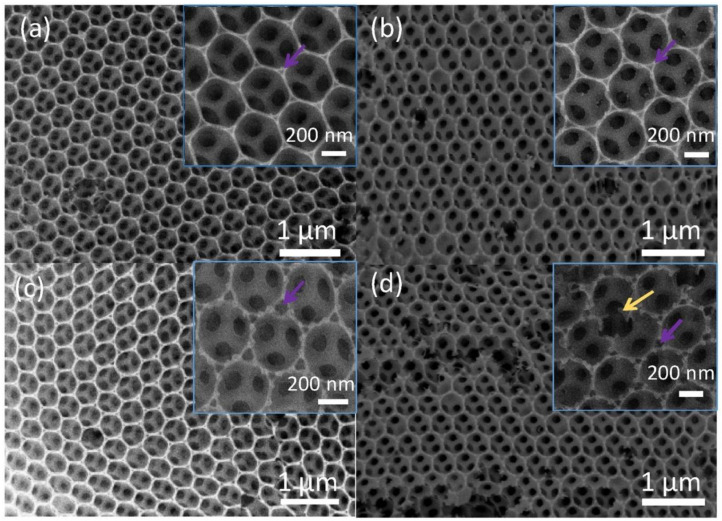
The SEM images of ZnO-Fe_3_O_4_ 3DIO samples with the Fe/Zn atom ratios of (**a**) 0:10, (**b**) 1:10, (**c**) 2:10, and (**d**) 3:10 samples [135]. Reprint permission was obtained from Elsevier.

In comparison to the dense and thick films, three-dimensional (3D) ordered composites have a large surface area, which guarantees the high number of adsorption sites, leading to higher sensing properties. Moreover, because of having a porous structure and open channels, surrounding gases can diffuse into the deep part of gas sensor, contributing to high gas response. In this context, the 3D inverse opal (3DIO) composite of ZnO-Fe_3_O_4_ was prepared using a template method [135]. The ratio of Fe/Zn atoms was varied to find the optimal gas sensor (Figure 10). For the gas sensor with a Fe:Zn atom ratio of 2:10 at 485 °C, a response (R_a_/R_g_) of 47–50 ppm acetone vapor was reported. It was also reported that the amount and size of mesoporous increased with the increase of Fe. As a result, the surface area increased and more gas molecules were able to be adsorbed on the surface of sensor. However, for the sensor with Fe:Zn, atomic ratio of 3:10, the structure was damaged and sensing properties decreased. Furthermore, ZnO/Fe_2_O_3_ heterojunctions significantly enhanced the sensing properties. ZnO/Fe_2_O_3_ heterojunctions were initially formed in air upon intimate contact between ZnO and Fe2O3. Subsequently, by exposure of the gas sensor to acetone vapor, the height of heterojunction barriers was decreased, leading to modulation of sensor resistance. This ultimately contributed to the gas response.

The two-dimensional (2D) heterostructure of the C_3_N_4_-SnO_2_ nanocomposite sensors with a high surface area of 57.13 m^2^/g were prepared for gas sensing studies [136]. The sensor showed a response (V_g_/V_a_) of 29–100 ppm acetone gas at a sensing temperature of 380 °C with fast response/recovery times (7 and 8 s, respectively). In addition, it was possible to detect as low as 67 ppb acetone, which is way below the exhaled breath concentration of diabetic people. Excellent acetone sensing properties were related to the exchange of electrons from SnO_2_ to C_3_N_4_ and formation of heterojunctions. Furthermore, the large surface area of C_3_N_4_ layer provided high number of adsorption sites for target gases. In addition, good selectivity of gas sensor was related to the large dipole moment of acetone arising from the C–C=O group of acetone. Dipole moment of acetone was higher than that of ethanol (1.69 D), methanol (1.70 D), NO (0.159 D), NO_2_ (0.316 D), NH_3_ (1.471 D), and CO (0.112 D) gases [113], leading to more interaction between acetone and the sensing layer.

**Figure 11:**
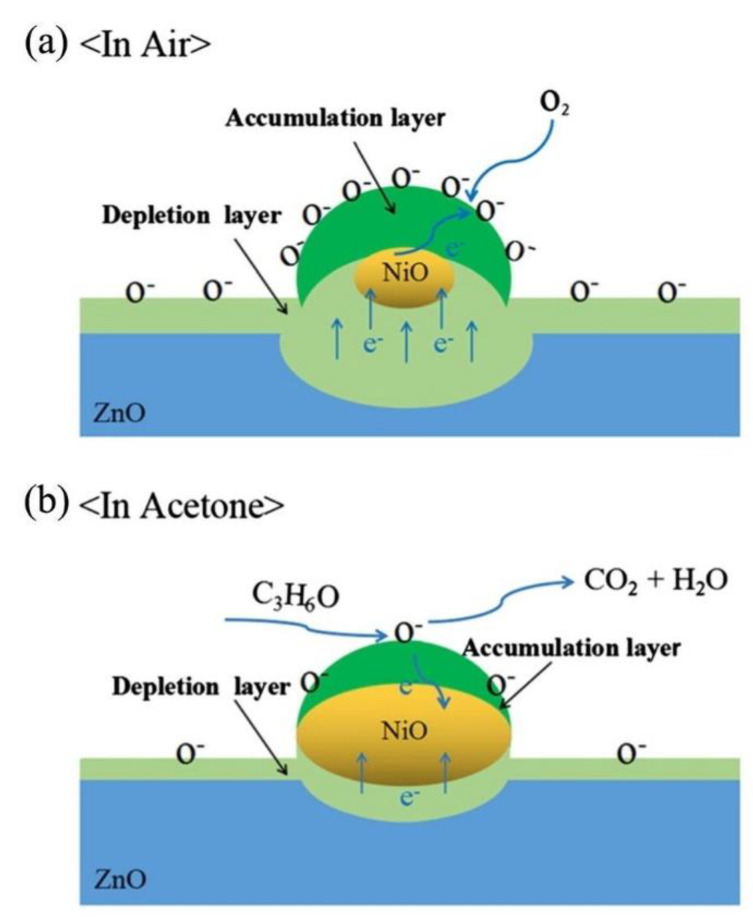
Mechanism of sensing for NiO-loaded ZnO in (**a**) air and (**b**) acetone [137]. Reprint permission was obtained from Elsevier.

NiO/ZnO composites were prepared by decoration of NiO NPs on the surfaces of ZnO hollow spheres using a solvothermal technique. The response (R_a_/R_g_) of NiO/ZnO composite sensor to 100 ppm acetone was ~29 at 275 °C, and both response and recovery times were 1 and 20 s, respectively. In dry air, the p–n junction between n-type ZnO and p-type NiO was produced. As a result, the resistance of the sensor was increased in air. In the acetone atmosphere, due to release of electrons to the surface of gas sensor, the resistance significantly decreased and a response appeared. Figure 11 schematically shows the sensing mechanism. Initially in air, electrons move from ZnO to NiO. During exposure to air, a depletion layer was developed on the surface of ZnO. On the other hand, due to abstraction of electrons from NiO, a hole accumulation layer was also created on the NiO [137]. Upon exposure to acetone gas, both thickness of the electron depletion layer and the hole accumulation layer were significantly decreased, leading to appearance of a sensing signal to acetone.

Defects and functional groups presenting on the graphene oxide (GO) surface act as high-energy adsorption sites for the gas molecules and can increase the response to acetone gas. In this regards, ZnO nanosheet/GO nanocomposites were prepared for detection of acetone [92]. The sensor with 10 wt% GO showed the highest sensing properties and a response (R_a_/R_g_) of 35.8–100 ppm acetone was observed at 240 °C. Presence of p-GO and n-ZnO potential barriers, high surface area of 2D nanocomposite, and presence of functional groups on the surface of GO were the main factors contributing to the sensor signal.

Organic–inorganic hybrid materials generally have enhanced sensing properties such as operating temperature and response time relative to pure materials due to a synergic effects between the different materials [138]. A polyaniline (PANI)/SnO_2_ hybrid material was prepared by a hydrothermal method for acetone sensing studies. The sensor revealed the highest response (R_g_/R_a_) of 1.68–800 ppm acetone gas at 60 °C. PANI is a p-type semiconductor and SnO_2_ an n-type, so that in intimate contacts, formation of p–n heterojunctions can create potential barriers in air. In acetone atmosphere, the height of potential barriers decreases, leading to sensing signal. However, the response was not compared with metal oxide nanocomposites [139].

Metal-organic frameworks-derived zinc oxide nanopolyhedra/S, N: graphene quantum dots/polyaniline (ZnO/S, N: GQDs/PANI) nanohybrid was synthesized by in situ polymerization route for acetone sensing applications. The sensor was able to work at room temperature and a response (R_a_/R_g_) of 1.33–0.5 ppm acetone with a fast response time of 15 s was reported. The presence of PANI in the nanohybrid led to the formation of p–n heterojunctions and also caused to a redistribution of charge carriers at the interface of n-type ZnO and p-type PANI/S, N: GQDs, decreasing the activation energy needed for the adsorption of acetone gas molecules. The presence of S, N: GQDs in the nanohybrid created Schottky contacts, which further enhanced the sensor response through effective capture and migration of electrons [140].

PPy-WO_3_ hybrid nanocomposites with different weight percentages of WO_3_ NPs (5–40 wt%) dispersed in polypyrrole (PPy) matrix were prepared for acetone sensing studies. PPy-WO_3_(20 wt%) sensor showed a fast, fairly sensitive, selective, and enhanced response toward acetone at 90 °C. The enhancement of acetone sensing properties of the PPy-WO_3_ hybrid nanocomposite film was related to the effective role of WO_3_ NPs in PPy matrix and formation of p–n hetrojunction region. The results demonstrate the potential application of PPy-WO_3_ hybrid sensor for noninvasive detection of acetone in breath [141].

Table 2 summarizes the acetone sensing properties of some metal oxide-based gas sensors reported in the literature. As expected, the response values of metal decorated, doped, and composite gas sensors were higher than those of the pristine acetone gas sensors listed in Table 1. This is because of greater resistance modulation in metal decorated, doped, and composite gas sensors relative to pristine gas sensors.

## 7. Advantages and Disadvantages of Different Metal Oxide Acetone Sensors

In general, pristine gas sensors have advantages of simple synthesis procedure and low overall fabrication cost. However, their sensing performance in term of response, selectivity, and sensing temperature is not comparable with doped, decorated, or composite gas sensors. This stem from few sources of resistance modulation in this type of gas sensors. Doped gas sensors also have simple synthesis procedures along with relatively low costs. Their performances are generally better than that of pristine gas sensors due to presence of defects and generally a finer morphology. However, their response is, in general, lower than decorated or composite gas sensors and, generally, we need to investigate optimal amount of doping to have the highest sensing performance. Decorated gas sensors have advantages such as relatively simple synthesis methods, high sensing performance, and selectivity can be enhanced by selection of appropriate material for decoration. However, if we use noble metal decoration, the overall cost of sensor increases, and like doped gas sensors, amount of noble metal decoration should be optimized on the surface of gas sensor. In addition, if the sensing temperature is high, the decorated noble metals may be sintered together or will be partially oxidized, leading to decrease of sensing performance. Composite gas sensors also have good sensing performances, especially in terms of sensing temperature, which can be significantly decreased relative to other gas sensors, but their composition should be optimized. Furthermore, explanation of exact sensing mechanism especially when ternary composite materials are used becomes difficult.

## 8. Conclusions and Outlook

In this review, different materials for realization of acetone gas sensors were discussed. It is well-known that sensing parameters such as sensitivity, response time, recovery time, and selectivity depend not only on sensing material but also on sensing surface conditions, sensor design, sensing layer morphology, sensing operation temperature, or a combination of different measurement strategies.

It was shown that not only pristine gas sensors are reliable for acetone sensors but also doped, decorated, and composite materials are widely used for acetone sensing studies. Most of these sensors were realized from simple binary metal oxides, even though ternary oxides such as ferrites and perovskites are also widely used for such application. The main sensing properties, namely, gas response, response and recovery times, and selectivity were mentioned for most of reported gas sensors in the review. Resistive-based gas sensors mainly based on metal oxides have not only low cost of synthesis and fabrication but also high stability over time, and most of them are not toxic for human beings. However, they generally need high temperatures for operation and accordingly consume high power.

Currently, big shortages of acetone sensors based on metal oxides are the lack of high sensitivity and selectivity and, relatively, high power consumption. In general, it is very difficult to realize a highly selective acetone sensor based on metal oxides. This is mostly due to similar nature of some VOCs, such as ethanol, with acetone. In general, there are some strategies to enhance selectivity of a metal oxide gas sensor towards acetone as follows: (i) tuning of sensing temperature as in general, different gases need different energies to overcome the adsorption barrier to be effectively adsorbed on the surface of gas sensor; (ii) noble metal decoration; (ii) use of a layer of metal organic frameworks (MOFs) with engineered porosities, which allow crossing of only certain gases; (iv) optimization of sensor chemical composition; and (v) use of metal oxides with ferroelectric properties, as acetone gas has a large dipole and can easily interact with ferroelectric metal oxides. A good strategy to reduce power consumption is the operation of gas sensor in self-heating mode, where the power consumption in range of microwatt can be attainable [146]. Furthermore, this weakness can be resolved using hybrid nanocomposites of metal oxides and organic materials that are able to work at room temperature. In fact, conducting polymers (CP) with advantages such as tunable conductivities and flexibilities in synthesis and processing can be used for detection of gases [147]. However, due to the high affinity of CPs toward VOCs and moisture present in the environment, they are sometimes unstable and show poor sensitivity and selectivity [148,149]. The use of hybrid nanocomposites based on CPs and metal oxides may result in room temperature gas sensors with improved and efficient gas sensing characteristics due to synergetic and complementary effects of both types of materials [150]. The hybrid p–n junction materials consisting of semiconducting oxides and CPs have been widely used to enhance the sensitivity of nanostructured sensors [151].

In future, with further advancements in the synthesis of low-cost and new hybrid materials with synergetic properties, it is expected that higher performance and more efficient acetone gas sensors (as compared with the present sensors) will be introduced. New functional materials with high interaction with acetone gas can facilitate adsorption of acetone on the surface of sensing layer. In addition, for better performance of gas sensor in humid environments, use of MOFs as coating layers on the surface of gas sensor with engineered porosities can increase the selectivity to acetone gas.

## Figures and Tables

**Table 1:** A summary of pristine acetone gas sensors reported in the literature.

Sensing Material	Acetone Concentration (ppm)	Sensing Temperature (°C)	Response (R_a_/R_g_) or (R_g_/R_a_)	Ref.
p-type gas sensors				
NiFe_2_O_4_ NPs	100	250	27.4	[18]
ZnCo_2_O_4_ NPs	200	200	38.2	[85]
BiFeO_3_ NPs	10	350	12	[74]
PrFeO_3_ NFs	200	180	141	[75]
Co_3_O_4_ nanosheet array	1000	111	36.5	[95]
Co_3_O_4_ nanocubes	500	240	4.9	[96]
n-type gas sensors				
Fe_2_O_3_ NPs	100	300	11.6	[88]
Fe_2_O_3_ NPs	100	340	9	[89]
ZnO NPs	100	230	33	[90]
ZnO NRs	100	300	32	[91]
Hollow ZnO NFs	100	220	70	[97]
Porous WO_3_ NFs	50	270	55.6	[98]
2D ZnO nanosheet	200	300	110	[92]

**Table 2:** A summary of decorated/loaded/composites acetone gas sensors reported in the literature.

Sensing Material	Acetone (ppm)	Sensing Temp. (°C)	Response (R_a_/R_g_) or (R_g_/R_a_)	Ref.
p-type gas sensors				
W-doped NiO hollow spheres	100	250	198.1	[101]
Yttrium-doped La_0.85_Y_0.25_MnO_3+δ_ NPs	500	300	26	[117]
n-type gas sensors				
SnO_2_/Au-In_2_O_3_ core–shell NFs	100	280	21	[107]
Rh-SnO_2_ NFs	50	200	60.6	[108]
Cr-doped ε-WO_3_ NPs	20	320	9	[113,114]
SnO_2_-Sm_2_O_3_ hierarchical structures	100	200	41.1	[121]
P-Co_3_O_4_ loaded-n-SnO_2_ NWs	50	300	62	[122]
Pt NPs -Fe_2_O_3_ nanocubes	100	139	25.7	[127]
PdAu-decorated SnO_2_ nanosheet	2	250	6.5	[128]
WO_2.72_(W_18_O_49_)/Ti_3_C_2_T_x_	5	300	4.2	[131]
Fe_2_O_3_/CuO	100	300	50	[134]
ZnO-Fe3O4	50	485	47	[135]
2D C_3_N_4_- SnO_2_ composite	100	380	29	[136]
NiO-loaded ZnO composite	100	275	29	[137]
In_2_O_3_/TiO_2_ NWs	10	250	33.3	[123]
1 wt% La_2_O_3_-doped ZnO NFs	100	300	34	[103]
Co-doped ZnO NFs	100	360	16	[104]
0.4 wt% Y-doped SnO_2_ hollow NFs	500	300	174	[105]
5 at% Ni-doped hollow SnO_2_ NFs	100	340	69.4	[105]
2 mol% Eu-doped SnO_2_ NFs	100	280	33	[106]
Rh_2_O_3_-decorated WO_3_ NFs	5	300	41.2	[124]
2D ZnO/GO nanocomposites	100	240	35.8	[92]
ZnO/S, N: GQDs/PANI	0.5	25	1.33	[140]
Pt-decorated In_2_O_3_ NPs	0.04	200	3.9	[142]
p-SmFeO_3_/n-ZnO nanocomposite	2	300	15	[143]
γ-Fe_2_O_3_/Al–ZnO nanocomposites	10	200	29	[144]
Gd-doped γ-Fe_2_O_3_	20	200	31.2	[145]
